# The reliability and validity of Child-to-parent Violence Questionnaire (CPV-Q) among Chinese adolescents

**DOI:** 10.1186/s41155-024-00314-1

**Published:** 2024-08-06

**Authors:** Weishi Xie, Mengxuan Wang, Linya Wang, Li Yang, Min Luo

**Affiliations:** 1 College of Education and Psychological Science, Hefei Normal University, 708, Xingzhi Building, No. 1688 Lianhua Road, Hefei, Anhui Province China; 2 Department of Psychology, Key Laboratory of Philosophy and Social Science of Anhui Province On Adolescent Mental Health and Crisis Intelligence Intervention, Hefei Normal University, Hefei, Anhui Province China

**Keywords:** Child-to-parent violence; Reliability; Validity; Adolescents

## Abstract

**Objective:**

To test the reliability and validity of the Chinese version of the Child-to-parent Violence Questionnaire (CPV-Q) in a group of Chinese adolescents.

**Methods:**

A total of 1138 adolescents (15.24 ± 1.17 years old) were tested with the Chinese version of CPV-Q, Parent-Adolescent Conflict Scale, and Adolescent Aggressive Behavior Scale of which 201 adolescents were retested 1 month later. The Chinese version of CPV-Q contains psychological, physical, financial, and control/domain factors with 14 items.

**Results:**

The four-factor model has good main fit indicators (father: *χ*^*2*^*/df* = 3.28, *CFI* = 0.96, RMSEA = 0.06; mother: *χ*^*2*^*/df* = 3.30, *CFI* = 0.96, RMSEA = 0.06); the scale has good criterion-related validity. The Cronbach’s *α* coefficients of the Chinese version of CPV-Q were 0.89 (father) and 0.88 (mother), and the Cronbach’s *α* coefficients of the four subscales were 0.81 ~ 0.84 (father) and 0.76 ~ 0.85 (mother). The test–retest reliability of the Chinese version of CPV-Q was 0.85 (father) and 0.83 (mother), and the test–retest reliability of the four subscales was 0.80 ~ 0.83 (father) and 0.75 ~ 0.84 (mother).

**Conclusion:**

Therefore, the CPV-Q has good reliability and validity for Chinese adolescents and can be used as an effective tool to evaluate Chinese adolescents’ violence toward their parents.

## Introduction

Child-to-parent violence (CPV) is a manifestation of child violence that occurs in the family environment and is considered as a “new” form of domestic violence (Zhang et al., 2018). It refers to a range of physical, psychological, and financial acts of aggression carried out by children against their parents in order to gain control over them (Cottrell, 2001). Due to the feelings of shame, embarrassment, or a desire to safeguard their children, parents often conceal their personal experiences from the public (Gabriel et al., 2018). The phenomenon of CPV has received relatively less attention in scholarly investigations when compared to other manifestations of domestic violence (Ulman & Straus, 2003; Zhang et al., 2019). In the Western countries, the prevalence of child-to-parent violence (CPV) spans a range of 3 to 27% (Holt, 2012), with a noted upward trend (Contreras et al., 2019, 2020; Edenborough et al., 2011). More specifically, instances of physical violence extend from 4.6 to 21% (Browne & Hamilton, 1998; Calvete et al., 2015; Ulman & Straus, 2003), while psychological violence is reported between 33 and 93% (Calvete et al., 2014; Ibabe & Bentler, 2016; Ibabe & Jaureguizar, 2013; Margolin & Baucom, 2014). Notably, Spain records the highest incidence of CPV, categorizing it among the most prevalent of crimes (Calvete & Pereira, 2019). Conversely, in China, the deeply ingrained cultural tenets of filial piety and the normative stance that family issues should remain confidential likely result in the underreporting of such occurrences. Empirical inquiries within China have illuminated a prevalence of physical violence against parents by their children at a rate of 8.3% and psychological violence at 36.8% (Zhang et al., 2019), figures which appear lower in comparison to Western counterparts (Beckmann et al., 2021; Contreras and Cano-Lozano, 2016; Margolin et al., 2014; Zhang et al., 2018; Zhang et al., 2019). In contemporary times, CPV not only disrupts the peaceful parent–child relationship (Clarke et al., 2017), but this complex social problem has many serious consequences, causing severe and enduring physical and psychological consequences for both parents and children.

During the period of adolescence, individuals experience significant physiological changes associated with puberty. Consequently, they are required to adapt to new educational environments or cope with enhanced academic pressure. In this context, inappropriate demands or guidance from mothers to their children may lead to increased parent–child conflict, which in turn increases the likelihood that children will become violent toward their parents (Zhang et al., 2018). Moreover, some studies have provided that the occurrence of psychological aggression perpetrated by teenagers toward their parents frequently serves as a precursor to acts of violence directed toward them (Zhang et al., 2019). Thus, Parent–Child Conflict and Adolescent Aggressive Behavior in this study are used as the criteria to assess the validity of the Child-to-parent Violence Questionnaire.

In order to conduct quantitative research on children’s violence against their parents, domestic and international researchers have developed the following scales: The Abused Parent Questionnaire (APQ) (for children with ADHD) from Ghanizadeh and Jafari (2010), Child to Parent Aggression Questionnaire (CPAQ) from Calvete et al. (2013), Child-to-Mother Violence Scale (CMVS) of, and the Explanations about Adolescent-to-parent Violence Scale (EEVFP: The Explanations about Adolescent-to-parent Violence Scale) (Cortina & Martín, 2021). Among them, the APQ evaluates four distinct categories of abuse from children with ADHD to their parents: physical, psychological, verbal, and financial. The CPAQ assesses both physical and psychological forms of aggression directed toward mothers and fathers. Additionally, it incorporates open-ended questions pertaining to the underlying causes of aggressive behavior. The CMVS measures the experiences and causes of children’s violence against their mothers, and the EEVFP assesses the causes of CPV from six dimensions. Additionally, expert domestic scholars Zhang Li et al. (2018) developed a questionnaire with a single-dimensional framework to assess various manifestations of physical violence perpetrated by adolescents against their mothers. However, despite the existence of various scales designed to assess CPV from different aspects, there remain the following shortcomings:

1. The pertinence and suitability of employing alternative scales for assessing CPV brings doubts
2. A single-dimensional scale cannot reflect the complexity of the structure of CPV
3. The only focus on evaluating children’s violence against their mothers fails to account for the evaluation of children’s violence toward their fathers
4. The scale above ignores the fact that children’s control over their parents is a crucial factor in CPV (Cottrell, 2001; Molla-Esparza & Aroca-Montolío, 2017)

Based on this, Contreras et al. (2019) developed the Child-to-parent Violence Questionnaire, which consists of 14 parallel questions assessing children’s violence against their fathers and mothers in four dimensions: psychological, physical, financial, and control/domain, including the reasons for aggression against parents. The scale has been shown to have good measurement characteristics in groups of children and adolescents in countries such as Spain and Chile (Contreras et al., 2019, 2020; Jimenez-García, 2020).

However, there is a lack of an appropriate measurement tool to investigate children’s violence against parents in the context of China. Hence, this study aimed to develop a scientifically rigorous and reliable measurement instrument for investigating children’s violence against parents by translating and revising the original CPV-Q into Chinese language and then analyzing the Chinese adaptation’s factor structure and psychometric characteristics.

## Material and methods

### Participants

According to the rules, the target sample size for CFA should be more than 10 times the number of items (Nunnally & Bernstein, 1994), so the minimum sample size required for this study is 140 subjects, and sample 1 intended for CFA meets this goal. The target sample size for correlation analysis is determined by G*Power before data collection (Faul et al., 2009). To meet the requirements of medium effect size *ρ* = 0.30 and power = 0.95 for correlation analysis, the minimum number of the required samples is 138 subjects. Both two samples (sample 1 with 1138 subjects, sample 2 with 201 subjects) in this study meet this goal.

We have recruited subjects through a convenient sampling method, and all the volunteers are middle school students hailing from three cities (Hefei, Huangshan, and Chuzhou) within the Anhui province of China. The survey was conducted in two phases: sample 1 and sample 2. Sample 1 contains 1138 students (640 males and 498 females; 408 middle school students and 730 high school students), who are aged between 9 and 18 (*M* = 15.24; *SD* = 1.17). The data of the individuals was divided into two cohorts using a random allocation method. One group of subjects (*n* = 569) was subjected to item and exploratory factor analysis to examine the scale. In contrast, the other group (*n* = 569) underwent validation factor analysis, internal consistency reliability analysis, and validity scale validity analysis of the instrument. After 1 month, 201 students (118 males and 83 females) aged between 9 and 18 (*M* = 15.24; *SD* = 1.17) in sample 1 were selected randomly for retesting as sample 2.

### Procedure

The present investigation has received approval from the Research Ethics Committee at Hefei Normal University. Before the research commenced, a meticulous process was undertaken to ensure the ethical and moral considerations were addressed. This included obtaining informed consent from both the parents or legal guardians and the relevant school authorities on behalf of all subjects participating in the study. Additionally, a rigorous review procedure was carried out to secure the necessary approvals for the participants’ involvement in the research, adhering to the strictest ethical guidelines and standards. This evaluation encompassed an assessment of the significance and objectives of the study as well as an acknowledgment of the voluntary and confidential nature of participation. Prior to completing the paper surveys during their class, the participants are provided with information regarding their right to withdraw from the study at any point. Moreover, they are given instructions to reply to all items in the questionnaire to make sure no data loss occurs in this study. The analysis of all the responses is conducted in a manner that guarantees the removal of any identifiable information.

### Measurements

#### The Chinese version of Child-to-parent Violence Questionnaire (CPV-Q)

With the explicit permission of the original authors of the instrument, back-translation was used in this study. Initially, one psychology professor and two postgraduate students in Psychology independently translated the English questionnaire into Chinese and discussed it repeatedly to form a first draft. After that, three psychology graduate students and two English graduate students translated the Chinese text into English again. Finally, the Chinese version of CPV-Q was formed after detailed discussions.

CPV-Q (Contreras et al., 2019) consists of four dimensions (psychological, physical, financial, and control/domain) with 14 items. CPV-Q is a validated instrument designed to assess child-to-parent violence behaviors, including the reasoning behind the aggression directed toward parents. The scale questionnaire is based on a 5-point Likert scale ranging from 0 to 4, where 0 indicates “never” (0 times), 1 indicates “rarely” (1 time), 2 indicates “sometimes” (2–3 times), 3 indicates “multiple times” (4–5 times), and 4 indicates “frequently” (more than 6 times).

#### Parent-Adolescent Conflict Scale (PACS)

The Parent-Adolescent Conflict subscale of the Parent-Adolescent Relationship Scale, developed by Dong and Lin (2011), was utilized. The Parent-Adolescent Conflict scale includes 3 items and a 5-point scale (1 for “never” to 5 for “always”). The higher the score, the more frequent conflicts between parents and children. In this study, Cronbach’s *α* was 0.75.

#### Adolescent Aggressive Behavior Scale (AABS)

The Adolescent Aggressive Behavior Scale (Dong & Lin, 2011) is a 10-item with a 4-point scale (1 for “never” to 4 for “always”), including two dimensions: physical and indirect aggression. The higher scores correspond to more aggressive behavior. In this study, Cronbach’s *α* was 0.82.

### Statistical analysis

The data analysis is carried out in two phases. In phase 1, to obtain the quality of each item, SPSS 24.0 is first used for item analysis on sample 1. Then, the exploratory factor analysis (EFA) is used to explore the structure of factors, as well as examine the factor loading of each item. Based on the above results, one determines whether any item needs to be deleted. In phase 2, AMOS 24.0 is used to conduct confirmatory factor analysis (CFA) on sample 2 so that we can verify the structure of the factor. Then, Pearson’s correlation was used to determine the correlation between CPV-Q and the criterion variables. Finally, the reliability of CPV-Q is evaluated by using Cronbach’s *α* and retest reliability. All data in this study do not contain missing data.

## Results

### Item analysis

Initially, the total scores of fathers and mothers on the Chinese version of the CPV-Q were calculated separately. These scores were arranged in decreasing order, and the top 27% and lowest 27% were identified as the high and low subgroups, respectively. Then, a *t*-test is conducted to examine the differences between the high and low subgroups for each item. The results showed significant differences between the two groups (all *p* < 0.001). In addition, we also calculated the item-total correlation of each item and found that there was a significant statistical correlation between the total score and all 14 items included in the questionnaire (father: *r*_(f)_ = 0.59 ~ 0.80, all *p*_(f)_ < 0.001; mother: *r*_(m)_ = 0.59 ~ 0.75, all *p*_(m)_ < 0.001). Hence, all items of the scale are retained.

### Construct validity

#### Exploratory factor analysis (EFA)

The Bartlett test of sphericity shows that KMO values were high for both fathers (0.90, *χ*^*2*^ = 4140.30, *p* < 0.001) and mothers (0.90, *χ*^*2*^ = 3741.62, *p* < 0.001), indicating that the data is suitable for EFA (Kaiser, 1974). This study employed principal component analysis and the varimax rotation method, and the extraction criterion was an eigen-root greater than one. As shown in Table 1, the results of this study confirmed the Chinese version of the CPV-Q with a four-factor structure of psychological, physiological, financial, and control/domain, which includes 14 different items. Father: The factor loading of each item ranges from 0.61 to 0.83, and the commonality ranges from 0.53 to 0.80. These values cumulatively explain 71.50% of the total variance. Mother: The factor loading of each item ranges from 0.63 to 0.82, and the commonality ranges from 0.60 to 0.82. These values cumulatively explain 69.32% of the total variance.

**Table 1:** Factor loading, commonality, and scores for each topic in the Chinese version of CPV-Q (*n* = 569)

Item	Father					Mother				
Number	1	2	3	4	Commonality	1	2	3	4	Commonality
T2	0.83				0.79	0.77				0.71
T3	0.8				0.74	0.75				0.62
T1	0.76				0.69	0.71				0.64
T4	0.61				0.53	0.73				0.61
T8		0.81			0.75		0.75			0.7
T11		0.78			0.8		0.82			0.8
T10		0.75			0.72		0.81			0.82
T7			0.81		0.79			0.75		0.8
T6			0.78		0.74			0.81		0.72
T12			0.77		0.72			0.81		0.75
T14				0.8	0.72				0.78	0.66
T9				0.73	0.66				0.74	0.63
T5				0.72	0.63				0.64	0.6
T13				0.71	0.74				0.63	0.65

1 = psychological; 2 = physical; 3 = financial; 4 = control/domain

#### Confirmatory factor analysis (CFA)

**Fig. 1:**
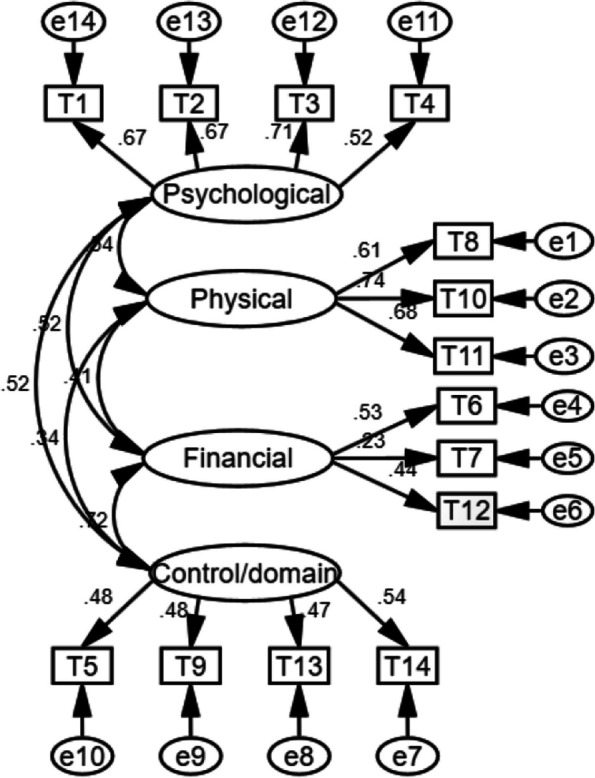
Diagram of the Chinese version of CPV-Q (father) four-factor structural equation model

**Fig. 2:**
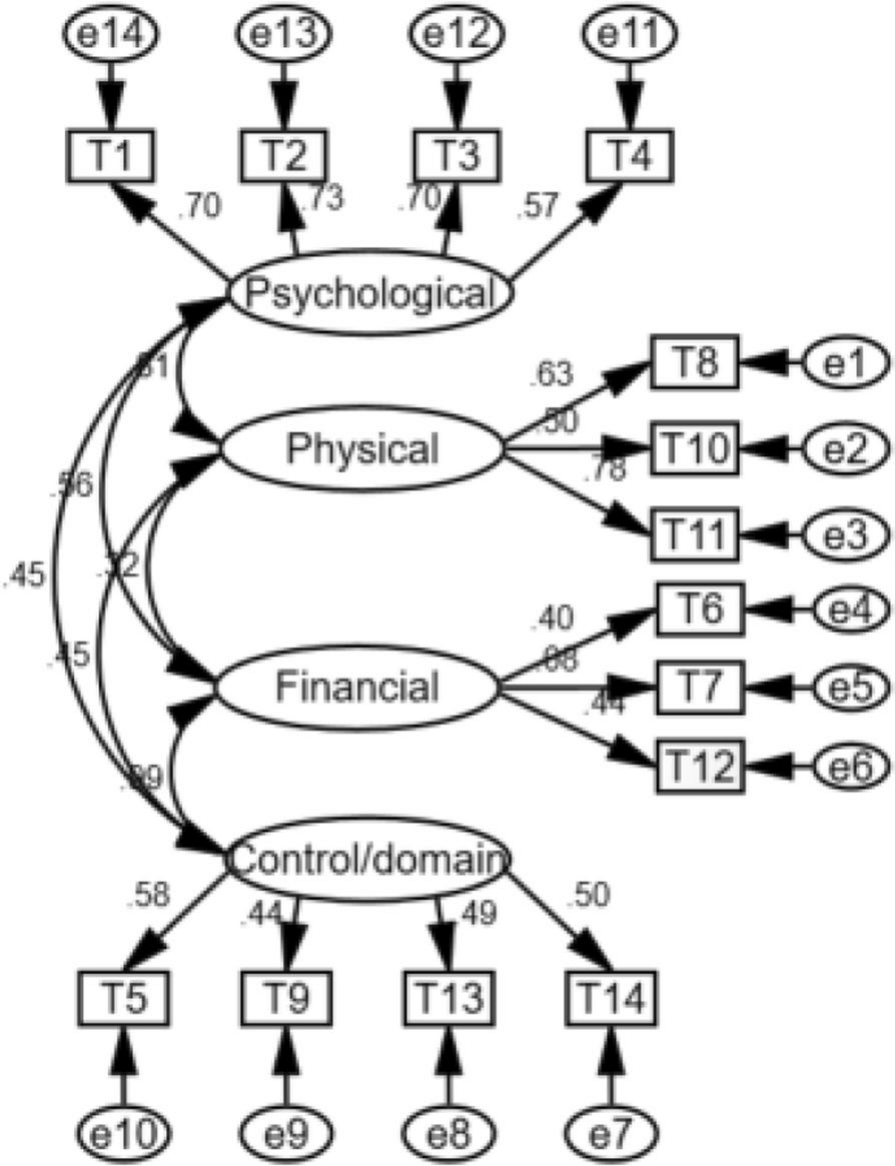
Diagram of the Chinese version of CPV-Q (mother) four-factor structural equation model

The remaining 50% of participants in sample 1 (*n* = 569) were used to conduct a validation factor analysis of the four-factor structural model of the CPV-Q Chinese version. The outcomes of the study demonstrate that the four-factor model index of the CPV-Q Chinese version meets the psychometric criteria. All of these showed that the Chinese version of the CPV-Q was significantly improved compared to the one-factor model, which indicated the equivalence between the Chinese version of CPV-Q and its prototype. The four-factor structural configuration is also graphically represented in Figs. 1 and 2 (Table 2).

**Table 2:** Fit index of the Chinese version of CPV-Q model (*n* = 569)

		χ^*2*^*/df*	RMR	RMSEA	CFI	GFI	NFI	TLI	IFI
Father	Single factor	15.26	0.02	0.16	0.73	0.74	0.72	0.68	0.73
	Four-factor	3.28	0.09	0.06	0.96	0.94	0.94	0.95	0.96
Mother	Single factor	13.01	0.02	0.15	0.75	0.77	0.74	0.7	0.75
	Four-factor	3.3	0.01	0.06	0.96	0.95	0.94	0.95	0.95

### Exploratory and confirmatory analyses of reasons for CPV

Bartlett’s test indicated that the correlations between items was not zero, *χ*^*2*^(28) = 0.884.53, *p* < 0.001. Furthermore, KMO was equal to 0.77, indicating that the correlation matrix was suitable for factorial analysis. The final solution with 8 items and two factors explained 53% of the variance. Factors 1 and 2 were designated as instrumental and reactive reasons, respectively. The CFA demonstrated that the model fit was not absolute, *χ*^*2*^(19) = 67.21, *p* < 0.001. The fit indexes obtained were adequate, *CFl* = 0.94, *TLl* = 0.92, *GFI* = 0.97, *RMR* = 0.01, RMSEA = 0.07. Cronbach’s *α* for factor 1 was 0.75, and it was 0.52 for factor 2. The correlation between factors 1 and 2 was 0.33 (see Table 3).

**Table 3:** Descriptive statistics and factorial loading of reasons for child-to-parent violence in the confirmatory analysis

	CFA		Descriptive statistics	
Item	IR	RR	M	SD
1	0.84		0.28	0.33
2	0.77		0.42	0.42
3	0.73		0.25	0.52
4	0.68		0.16	0.48
5		0.66	0.67	0.97
6		0.64	0.30	0.66
7		0.60	0.33	0.21
8		0.72	0.31	0.63

*IR *Instrumental reasons, *RR *Reactive reasons

### Criterion-related validity

Correlation analyses have been conducted for the Chinese version of CPV-Q as well. As Table 4 shows, they are significantly positively correlated with the score of parent–child conflict and aggression behavior. Their correlation coefficients are 0.30 and 0.40 respectively.

**Table 4:** Correlation of the Chinese version of CPV-Q with scores on each validity scale (*n* = 569)

	M ± SD	CPV-Q (father)	CPV-Q (mother)	PC	AB
CPV-Q (father)	0.20 ± 4.57	1			
CPV-Q (mother)	0.18 ± 4.30	0.69***	1		
PC	1.54 ± 1.31	0.30***	0.405***	1	
AB	1.16 ± 2.83	0.40***	0.39***	0.34***	1

Note: *p＜0.05,**p＜0.01,***p＜0.001

### Reliability

#### Internal consistency

Cronbach’s *α* is used to evaluate the internal consistency of the scale. The results show that Cronbach’s *α* of the Chinese version of CPV-Q are 0.89 for fathers and 0.88 for mothers. In terms of the four dimensions, the fathers have Cronbach’s *α* coefficients within a range of 0.81–0.84, whereas the mothers’ results ranged from 0.76 to 0.85.

#### Test–retest reliability

The retest data obtained from 201 participants after 1 month indicated that the Chinese version of CPV-Q is reliable: 0.85 for fathers and 0.83 for mothers; the retest reliability of the four dimensions ranged between 0.80 and 0.83 for fathers and 0.75 and 0.84 for mothers (Table 5).

**Table 5:** Reliability coefficients of the Chinese version of CPV-Q and the subscales

	Father		Mother	
	Cronbach’s *α*	Test–retest reliability	Cronbach’s *α*	Test–retest reliability
Summary scale	0.89	0.85	0.88	0.83
Psychological	0.84	0.8	0.81	0.79
Physical	0.83	0.83	0.85	0.84
Financial	0.84	0.82	0.82	0.8
Control/domain	0.81	0.8	0.76	0.75

## Discussion and conclusions

Currently, research on the prevention and intervention of CPV in China is still insufficient because few measurement tools are available for adolescents. Given that adolescents are in a stage of development characterized by significant physical and psychological changes, they are concurrently cultivating a sense of autonomy and fostering a desire to transcend parental oversight. Consequently, it is plausible that conflicts with parents may escalate appreciably during this developmental phase (Gallagher, 2004). In addition, parent–child conflict may have a direct negative impact on the cognitive, emotional, and interpersonal well-being of children (Hess, 2022). Therefore, it is necessary to compile or revise a measurement tool in China to evaluate violence against parents among adolescents, which can also provide an assessment tool for effective prevention and intervention. This study strictly follows the standardized translation process, develops the Chinese version of the CPV-Q, and tests its reliability and validity.

The modified Chinese version of CPV-Q is similar in structure to the original questionnaire. It encompasses a total of 14 items, which are categorized into four distinct factors: psychological, physical, financial, and control/domain. The findings from the item analysis reveal statistically significant differences between the subgroups with high and low total scores on the Chinese CPV-Q, across all 14 questions that were examined. Moreover, the correlation coefficients between the scores of these items and the overall score exceeded 0.59, indicating statistical significance. It indicates that the Chinese version of CPV-Q has good construct validity. Firstly, the results of the EFA showed that its structure closely matched that of its original scale (Contreras et al., 2019). Regarding to this version of CPV-Q, the fit indicators of the four-factor model demonstrated well. Moreover, the findings of the CFA also revealed that the fit indexes for the four-factor model surpassed the acceptable level.

The Chinese version of the CPV-Q also has good criterion-related validity. In this study, the Chinese version of CPV-Q is positively correlated with parent–child conflict and aggressive behavior. It indicates that the Chinese version of CPV-Q has good construct validity in the Chinese adolescent population. The findings of this study are in alignment with prior research (Jimenez-Garcia et al., 2020) and are congruent with the conceptualization of CPV as delineated by Contreras et al. (2019). The manifestation of CPV is multifaceted, encompassing psychological, physical, financial, and control/domain dimensions. A spectrum of individual characteristics, such as gender, age, personality, psychological makeup, exposure to family violence, parenting style, peer and academic influences, cultural backdrop, and various other correlates, has been linked to the manifestation of CPV. The presence of a detrimental factor within an individual’s environment or experience can enhance their vulnerability to the adverse effects of other risk factors, a phenomenon described as “risk magnification.” The synergistic influence of these risk factors exerts a more pernicious impact than any single factor in isolation, as demonstrated by Cano-Lozano et al. (2021).

The reliability analyses indicate that the Cronbach’s *α* reliability of the Chinese version of CPV-Q and the retest reliability after an interval of 1 month are both good. Cronbach’s *α* of the CPV-Q of the father and the CPV-Q of the mother are 0.89 and 0.88, respectively. Cronbach’s *α* reliability for the four dimensions ranges from 0.81 to 0.84 (fathers) and from 0.76 to 0.85 (mothers). One month later, the retest reliability of the Chinese version of CPV-Q is 0.85 (fathers) and 0.83 (mothers), and the retest reliability of the four dimensions is 0.80 to 0.83 (fathers) and 0.75 to 0.84 (mothers). This result obeys the psychometric criteria, which means that the Chinese version of CPV-Q has good internal consistency and stability.

In short, the factor structure of the Chinese version of CPV-Q, as examined in a sample of Chinese adolescents, is consistent with the structure presented in prior international research. All the indicators meet the psychometric standards, exhibiting good reliability and validity. The Chinese version of CPV-Q can suggest a scientific evaluation tool for assessing CPV among the adolescent population of China.

We translate the CPV-Q into a Chinese scale and conduct a rigorous psychometric assessment. Overall, the results suggest that the CPV-Q is an effective tool to measure changes in children’s violence against parents among Chinese adolescents. Based on the theory of the transtheoretical model, the development of scale is considered an indispensable prerequisite for any attempt to implement and evaluate health education and to promote prevention and intervention (Sarbandi et al., 2013). Thus, the results of this study can be well applied to the prevention and intervention of children’s violence against parents among Chinese adolescents.

Several limitations should be highlighted. Firstly, the volunteers were middle school students from only three cities in China, which may not be fully representative of the entire Chinese youth group. Secondly, the volunteers were not recruited randomly, which may limit the generalizability of the results. Thirdly, although the survey is anonymous, socio-cultural factors may still influence the obtained data. Despite these limitations, the present study provides reliable evidence of the psychometric properties of CPV-Q among Chinese adolescents.

## Conclusion

This study has examined the factor structure, reliability, and standard correlation validity of the CPV-Q in Chinese adolescents. The results indicate that the Chinese version of CPV-Q has good psychometric properties in China. Particularly, the Chinese version of CPV-Q has a clear factor structure with an acceptable fit index. The Cronbach’s *α* coefficient and retest reliability of the Chinese version of CPV-Q are high. The Chinese version of CPV-Q is positively correlated with parent–child conflict, aggressive behavior. It indicates that the Chinese version of CPV-Q has high criterion correlation validity, suggesting that the Chinese version of CPV-Q is a valid and reliable tool to assess children’s violence against parents among Chinese adolescents.

## Abbreviations

CPV-Q: Child-to-parent Violence Questionnaire
APQ: The Abused Parent Questionnaire
CPAQ: Child to Parent Aggression Questionnaire
CMVS: Child-to-Mother Violence Scale
EEVFP: The Explanations about Adolescent-to-parent Violence Scale
PCCS: Parent-Child Conflict Scale
AABS: Adolescent Aggressive Behavior Scale
EFA: Exploratory factor analysis
CFA: Confirmatory factor analysis
KMO: Kaiser-Meyer-Olkin
RMR: Root mean square residual
CFI: Comparative fit index
RFI: Relative fit index
GFI: Goodness-of-fit index
TLI: Tucker-Lewis index
IFI: Incremental fit index
RMSEA: Root mean square error of approximation
